# Upper zone of growth plate and cartilage matrix associated protein protects cartilage during inflammatory arthritis

**DOI:** 10.1186/s13075-018-1583-2

**Published:** 2018-05-02

**Authors:** Fritz Seuffert, Daniela Weidner, Wolfgang Baum, Georg Schett, Michael Stock

**Affiliations:** Department of Internal Medicine 3—Rheumatology and Immunology, Friedrich-Alexander-University Erlangen–Nürnberg (FAU), Universitätsklinikum Erlangen, 91054 Erlangen, Germany

**Keywords:** Arthritis, Cartilage degeneration, Osteophyte formation, Aggrecanase, ADAMTS

## Abstract

**Background:**

ADAMTS aggrecanases play a major role in cartilage degeneration during degenerative and inflammatory arthritis. The cartilage-specific secreted protein Upper zone of growth plate and cartilage matrix associated protein (Ucma) has been shown to block ADAMTS-triggered aggrecanolysis in experimental osteoarthritis. Here we aimed to investigate whether and how Ucma may affect cartilage destruction and osteophyte formation in the context of inflammatory arthritis.

**Methods:**

Ucma–ADAMTS5 protein interactions were studied using slot blot and solid phase binding assays. Chondrocyte cultures were stimulated with ADAMTS5 or IL-1β in the presence or absence of Ucma and aggrecanolysis was assessed by neoepitope formation. Arthritis was induced by transfer of K/BxN serum into wild-type (WT), Ucma-deficient and WT mice treated with recombinant Ucma. Cartilage proteoglycan loss and cartilage damage was assessed by safranin-O stain, aggrecanase-induced neoepitope formation and histomorphometry, respectively. Osteophytes were assessed by histomorphometry, micro-computed tomography, RNA in-situ hybridisation for *collagen10a1* and *osteocalcin*, and staining for TRAP activity. Gene expression analyses were performed using real-time RT-PCR.

**Results:**

Ucma physically interacted with ADAMTS5 and blocked its aggrecanase activity in chondrocyte cultures. Ucma was highly expressed in the articular cartilage and in osteophytes during arthritis. Ucma had no effect on inflammation and bone erosion. In contrast, Ucma-deficient mice developed significantly more severe cartilage proteoglycan loss and cartilage destruction. Conversely, treatment with Ucma inhibited cartilage degeneration in arthritis. Ucma effectively inhibited ADAMTS5-triggered or IL-1β-triggered aggrecanolysis in vitro and in vivo. Furthermore, osteophyte formation was reduced in Ucma-deficient mice.

**Conclusions:**

These results indicate that Ucma inhibits aggrecanolysis by physical interaction with ADAMTS5 and protects from cartilage degeneration in inflammatory arthritis. Ucma therefore represents an interesting novel and specific target for preventing cartilage degradation in the context of inflammatory arthritis.

**Electronic supplementary material:**

The online version of this article (10.1186/s13075-018-1583-2) contains supplementary material, which is available to authorized users.

## Background

Chronic inflammatory arthritides, such as rheumatoid arthritis (RA) and psoriatic arthritis (PsA), lead to substantial changes in the articular cartilage. On the one hand there is net loss of articular cartilage since synovitis induces the release of factors that inhibit essential pathways for matrix synthesis by chondrocytes and trigger catabolic pathways such as the expression of matrix-degrading enzymes [1]. Interleukin-1 (IL-1) and tumour necrosis factor alpha (TNFα), for instance, are potent cartilage-degrading factors released from the synovial membrane, which induce chondrocyte dedifferentiation and matrix breakdown. The latter is guided by the excessive release of matrix-degrading enzymes such as members of the A disintegrin and metalloproteinase with thrombospondin motifs (ADAMTS) and matrix metalloproteinase (MMP) families [2–4].

On the other hand, arthritis can also lead to local cartilaginous proliferations. In this case, osteo-chondroprogenitor cells in the periosteum start to proliferate and form condensations. These structures then undergo chondrogenic differentiation with deposition of cartilage matrix and form a template for ossification. The innermost chondrocytes mature towards hypertrophic chondrocytes, secreting a matrix rich in type X collagen, which becomes mineralised and eventually replaced by bone [5]. In this process, hypertrophic chondrocytes may also transdifferentiate into osteoblasts [6].

Apart from external factors such as cytokines, which affect cartilage homeostasis during arthritis, we hypothesised that there are factors which are produced by the cartilage itself and regulate cartilage homeostasis during arthritis. We previously identified the secreted Upper zone of growth plate and cartilage matrix associated protein (Ucma; also termed Gla-rich protein (GRP)) in a screen for cartilage-specific genes [7]. Ucma expression in mice is confined to cartilage, although it also migrates through the matrix and gets released from the cartilage compartment [7–10]. Knock-down of Ucma in zebrafish results in cartilage defects and a severely disturbed craniofacial development [11], while Ucma-deficient mice developed normally, without any overt alterations in cartilage and skeletal development [9]. Ucma-deficient mice, however, develop more severe experimental osteoarthritis after destabilisation of the medial meniscus (DMM), indicating a chondroprotective effect of Ucma [8].

Although of different etiopathogenesis, the terminal pathways leading to cartilage degeneration in osteoarthritis and inflammatory arthritis may be similar. In this study, we therefore investigated the nature of Ucma-dependent aggrecanase inhibition and the effect of Ucma on cartilage homeostasis during inflammatory arthritis. In our experiments we show that Ucma directly inhibits aggrecanase activity by physical interaction with ADAMTS5. Moreover, we demonstrate that Ucma-deficient mice developed more severe arthritis-triggered cartilage degradation, while they did not differ from wild-type mice in terms of clinical signs of arthritis and the extent of synovial inflammation. On the other hand, treatment with recombinant Ucma ameliorated cartilage degeneration during arthritis, suggesting that Ucma has protective effects on the cartilage. Ucma was not only expressed in articular cartilage but was also found in osteophytes. Ucma deficiency also affected the development of osteophytes with fewer osteophytes, osteoblasts and osteoclasts in Ucma-deficient mice during serum-induced arthritis, reflecting Ucma-dependent regulation of osteoblasts and/or osteoclasts, which are required for osteophyte formation and growth.

## Methods

### Protein interaction studies

Recombinant His-FLAG-tagged Ucma was expressed episomally in HEK293EBNA cells and purified from conditioned medium by affinity chromatography on nickel–nitrilotriacetic acid Sepharose (Qiagen), as reported previously [7]. Collagens type I and II (derived from chicken by pepsin extraction) and recombinant human ADAMTS5 (R&D Systems) were immobilised on PVDF membranes by vacuum blotting. Binding of recombinant Ucma to immobilised proteins was detected as described previously [8]. Briefly, blots were blocked with BSA and incubated in BSA or recombinant Ucma (100 ng/μl). Bound Ucma was detected using rabbit anti-Ucma (UCMA-1; 1:1000) or mouse anti-FLAG (1:1000; Sigma-Aldrich) antibody, anti-rabbit-IgG-HRP or anti-mouse-IgG-HRP antibody, and ECL-based chemoluminiscence.

For solid phase binding assays, ELISA plates were coated with BSA (blanks), collagen I, collagen II or ADAMTS5 (2–250 ng/well, respectively). As reported previously, plates were blocked with BSA and incubated with Ucma (100 ng/ml). Bound Ucma was detected using mouse-anti-FLAG antibody (1:1000), anti-mouse-IgG-HRP antibody and o-phenylenediamine-based colorimetric detection at 492 nm [8]. Incubation with BSA instead of Ucma did not reveal significant signals.

### Cell culture

Chondrogenic 4C6 cells were maintained in DMEM/Ham’s F12 medium (Gibco®, ThermoFisher Scientific) with 10% fetal calf serum (FCS), as described previously [12]. For chondrogenic maturation and matrix production, cells were cultured confluently for 7 days in DMEM/Ham’s F12 medium with 10% FCS, 10 mM β-glycerophosphate and 50 μg/ml ascorbate. Subsequently, cells were stimulated with indicated doses of recombinant ADAMTS5, IL-1β or Ucma for 24 h in serum-free DMEM/Ham’s F12 medium before immunohistochemical detection of NITEGE epitopes. The NITEGE-positive chondrocyte culture area was quantified using ImageJ software (*n* = 3 or 4).

### Mice and induction and scoring of serum-induced arthritis

C57BL/6 mice were obtained from Elevage Janvier. We have previously generated Ucma-deficient mice. These mice and their wild-type (WT) littermates were bred in-house under specific pathogen-free conditions. Significant abnormalities in growth, fertility, development or skeletal morphology were not detected in Ucma-deficient mice and they were indistinguishable in size and weight from WT C57/B6 mice [9]. Experimental arthritis in WT and Ucma-deficient mice was induced by transfer of 100 μl K/BxN serum to recipient mice via intraperitoneal (i.p.) injection (serum-induced arthritis (SIA), *n* = 6–8 per group.). Serum was obtained from 8-week-old K/BxN mice, as reported previously [13, 14]. Ucma treatment of C57BL/6 mice during SIA was performed by daily i.p. injections, each with 100 μl PBS or 2 μg recombinant Ucma in 100 μl PBS, starting at day 1 after serum transfer (*n* = 3–5 per group). Clinical signs of arthritis, including loss of grip strength (0 = normal grip strength, −1 = mildly reduced, −2 = moderately reduced, −3 = severely reduced and −4 = no grip strength) and paw swelling (0 = no swelling, 1 = detectable, 2 = mild, 3 = moderate and 4 = severe swelling of toes and ankle), were assessed in a blinded manner at the indicated time points, as described previously [14, 15]. Ten or 14 days after serum transfer, the mice were sacrificed. The left hind paw of each mouse was used for serial paraffin sections and the right hind paw was used for micro-computed tomography (micro-CT) or gene expression studies. All experiments were performed with the approval of the local ethics authorities (Government of Mittelfranken, Ansbach, Germany) and according to the regulations of the animal facilities in Germany.

### Paw histology

Sections were stained with hematoxylin and eosin (H&E) for the analysis of inflammation, osteophyte formation and bone erosion or with safranin O for the assessment of proteoglycan content of articular cartilage. Osteoclast counts were determined on sections after histochemical detection of tartrate-resistant acid phosphatase (TRAP) using the leukocyte acid phosphatase staining kit (Sigma-Aldrich). All histological quantifications were carried out in a blinded manner by histomorphometry using ImageJ software or an OsteoMeasure system (OsteoMetrics), as reported previously [8, 15]. All analyses were performed on two or three comparable central sections per paw.

### Micro-computed tomography imaging and analysis

Micro-CT image acquisition and analysis of hind paws was performed as reported recently [16]. Briefly, calcified tissue visualisation was performed using the cone-beam Desktop Micro Computer Tomograph “μCT 40” (SCANCO Medical AG, Bruettisellen, Switzerland) at 55 kVp, 145 μA and 200 ms integration time with the operating system “Open VMS” (SCANCO).

### Immunodetection of Ucma and ADAMTS-mediated aggrecan cleavage products

Immunohistological detection of Ucma on paw sections from mice was carried out after hyaluronidase-mediated antigen retrieval with UCMA ab-2 antibody (1:500), SignalStain® Boost IHC Detection Reagent (HRP rabbit; Cell Signaling Technologies) and DAB as described recently [8, 9]. Similarly, ADAMTS-generated aggrecan cleavage products containing the NITEGE neoepitope were detected using a polyclonal rabbit anti-NITEGE antibody (Ab1320, 1:1000; IBEX Pharmaceuticals Inc.). Serum levels of Ucma were detected by spotting 10 μl serum from PBS-treated or Ucma-treated mice onto a nitrocellulose membrane. After blocking in 5% BSA, Ucma was detected using UCMA1 antibody (1:1000) and anti-rabbit IgG [9].

### RNA in-situ hybridisation

RNA in-situ hybridisation on paw sections was carried out with digoxigenin-labelled antisense riboprobes as outlined previously [9, 17]. Briefly, antisense riboprobes for *collagen 10 a1* (*Col10a1*; nucleotides 1689–2114 in NM_009925) and *osteocalcin* (nucleotides 8–378 in X04142.1) were prepared by in-vitro transcription in the presence of digoxigenin-labelled dNTPs (Roche), as reported previously [17]. Rehydrated paw sections were hybridised with antisense riboprobes at 55 °C overnight. After stringent washing, bound riboprobes were detected with an alkaline phosphatase-conjugated anti-digoxigenin antibody and BM Purple (Roche) detection reagent.

### Gene expression analysis

Total RNA was extracted from joint tissue in the paw (*n* = 3/group), using the RNeasy Fibrous Tissue Mini Kit (Qiagen). cDNA was synthesised using SuperScript II reverse transcriptase (Invitrogen) and mRNA expression relative to *cyclophilin A* was quantified by real-time RT-PCR using gene-specific primers (Additional file 1), as described previously [7].

### Statistical analysis

Data are presented as the mean ± standard error of the mean. Statistical significance was evaluated by one-way analysis of variance (ANOVA) and Tukey’s multiple comparison test after confirming normal distribution using the Kolmogorov–Smirnov test. Statistical analysis was performed using Graph-Pad Prism software.

## Results

### Ucma physically interacts with ADAMTS5 and blocks its aggrecanase activity in cultured chondrocytes

**Fig. 1:**
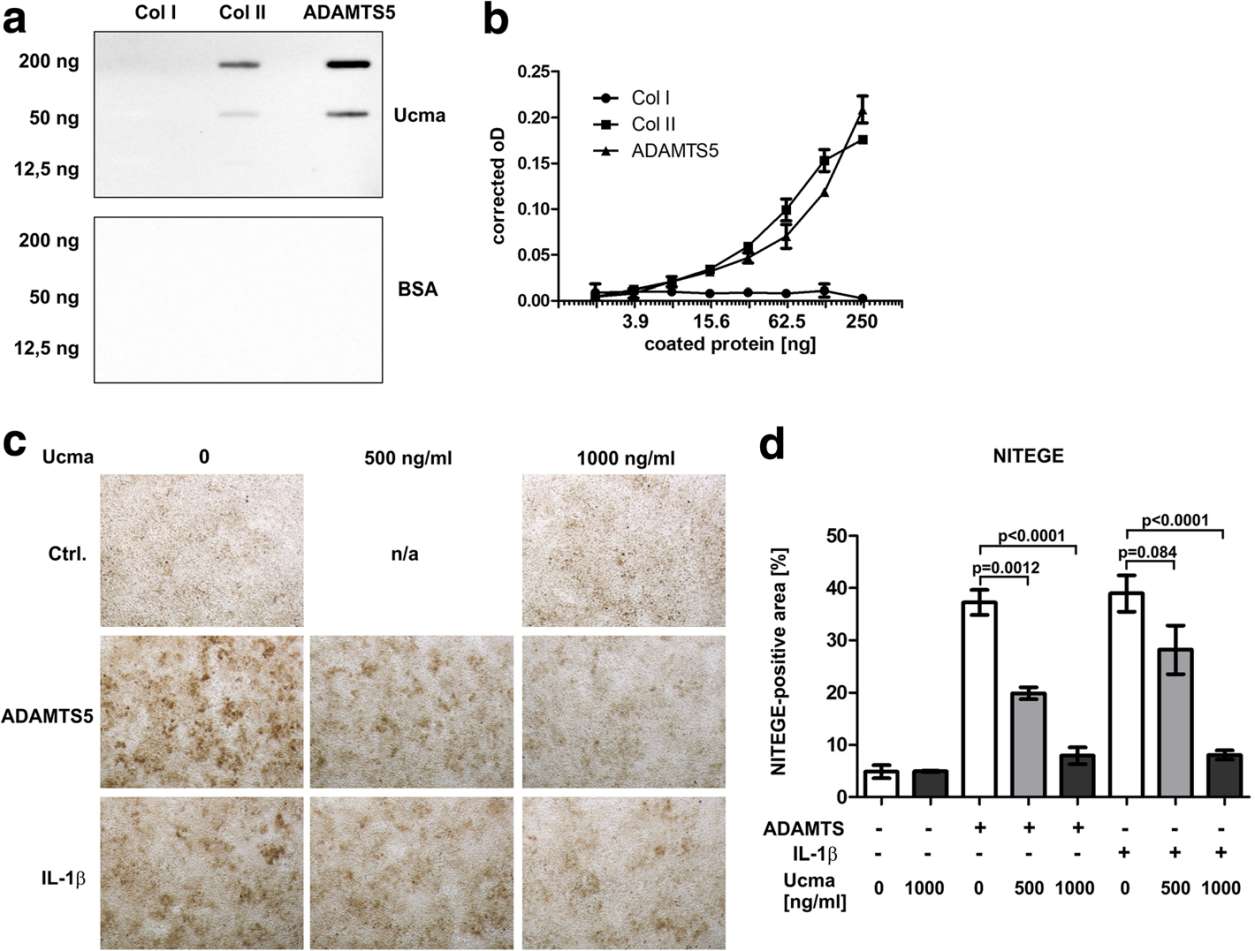
Ucma physically interacts with ADAMTS5 and inhibits its aggrecanase activity in chondrocyte cultures. **a** Ucma–ADAMTS5 interactions investigated by slot blot binding assays: indicated amounts of recombinant ADAMTS5 blotted onto PVDF membrane incubated with recombinant FLAG-tagged Ucma (upper panel) or BSA (lower panel) and bound Ucma detected with anti-FLAG antibody and anti-mouse IgG-HRP. Collagen I and II blotted as negative and positive controls, respectively. **b** Solid phase assay to detect Ucma–ADAMTS5 interactions. ADAMTS5 and type I and type II collagen coated to multi-well ELISA plates at indicated doses and then incubated with Ucma. Detection of bound Ucma performed with anti-FLAG antibody and colorimetric detection system. Ucma protein interactions represented as oD at 492 nm corrected by oD_492_ of BSA-coated wells. **c, d** Chondrogenic 4C6 cell cultures treated with or without Ucma (doses indicated) and recombinant ADAMTS (20 nM) or IL-1β (2.5 ng/ml) for 24 h in serum-free medium and stained for ADAMTS-specific aggrecan cleavage product (NITEGE). NITEGE-positive chondrocyte culture area quantified using ImageJ software (**d**). Means ± SEM shown; *n* = 3 or 4. Representative data from three independent experiments each. Coll collagen, ADAMTS A disintegrin-like and metalloproteinase domain with thrombospondin-1 repeats, BSA bovine serum albumin, Ctrl. control, IL interleukin, n/a not available, NITEGE ADAMTS-specific aggrecan neoepitope, Ucma Upper zone of growth plate and cartilage matrix-associated protein

We have demonstrated previously that Ucma blocks ADAMTS-specific aggrecanase activity in vitro [8]. In order to identify the mechanism of Ucma-mediated aggrecanase inhibition, we investigated protein–protein interactions between Ucma and ADAMTS5. Therefore, indicated doses of recombinant ADAMTS5 were immobilised on PVDF membranes. Collagen I and collagen II as negative and positive controls, respectively, were also blotted onto the same membranes. Blots were then incubated with recombinant FLAG-tagged Ucma or BSA. Detection of bound Ucma using anti-FLAG (Fig. 1a) or anti-Ucma (Additional file 2) antibody revealed that Ucma directly interacts with ADAMTS5. As expected, Ucma also bound to collagen II but not to collagen I (Fig. 1a) [8]. Solid phase binding assays using ADAMTS5-coated or collagen-coated ELISA plates and Ucma in the liquid phase confirmed binding of Ucma to ADAMTS5 (Fig. 1b). These findings imply that Ucma physically interacts with ADAMTS5 and thereby blocks the aggrecanase activity of ADAMTS5.

In order to test the feasibility of excess Ucma doses to control aggrecanase activities in cartilage under inflammatory conditions, we investigated the effect of recombinant Ucma on ADAMTS-specific aggrecan neoepitope (NITEGE) formation in cultured chondrocytes. Therefore, chondrogenic 4C6 cells were treated with recombinant ADAMTS5 in the absence or presence of Ucma. Afterwards, NITEGE formation in the chondrocyte layer was determined immunohistochemically. As expected, ADAMTS5 induced the extracellular formation of NITEGE-containing aggrecan cleavage products in 4C6 cultures. UCMA, however, inhibited ADAMTS5-induced NITEGE formation in a dose-dependent manner (Fig. 1c, d), confirming Ucma-dependent inhibition of ADAMTS5 aggrecanase. Intriguingly, recombinant Ucma also blocked IL-1β-induced NITEGE formation in 4C6 chondrocyte cultures (Fig. 1c, d). NITEGE formation was hardly detectable in untreated (no ADAMTS5, no IL-1β) 4C6 cultures and Ucma did not affect NITEGE formation under these conditions (Fig. 1c, d). This suggests that excessive Ucma might be feasible for control of pathologic ADAMTS activities in cartilage, which can be found under inflammatory conditions.

### Ucma is expressed in the articular cartilage and in osteophytes during arthritis

**Fig. 2:**
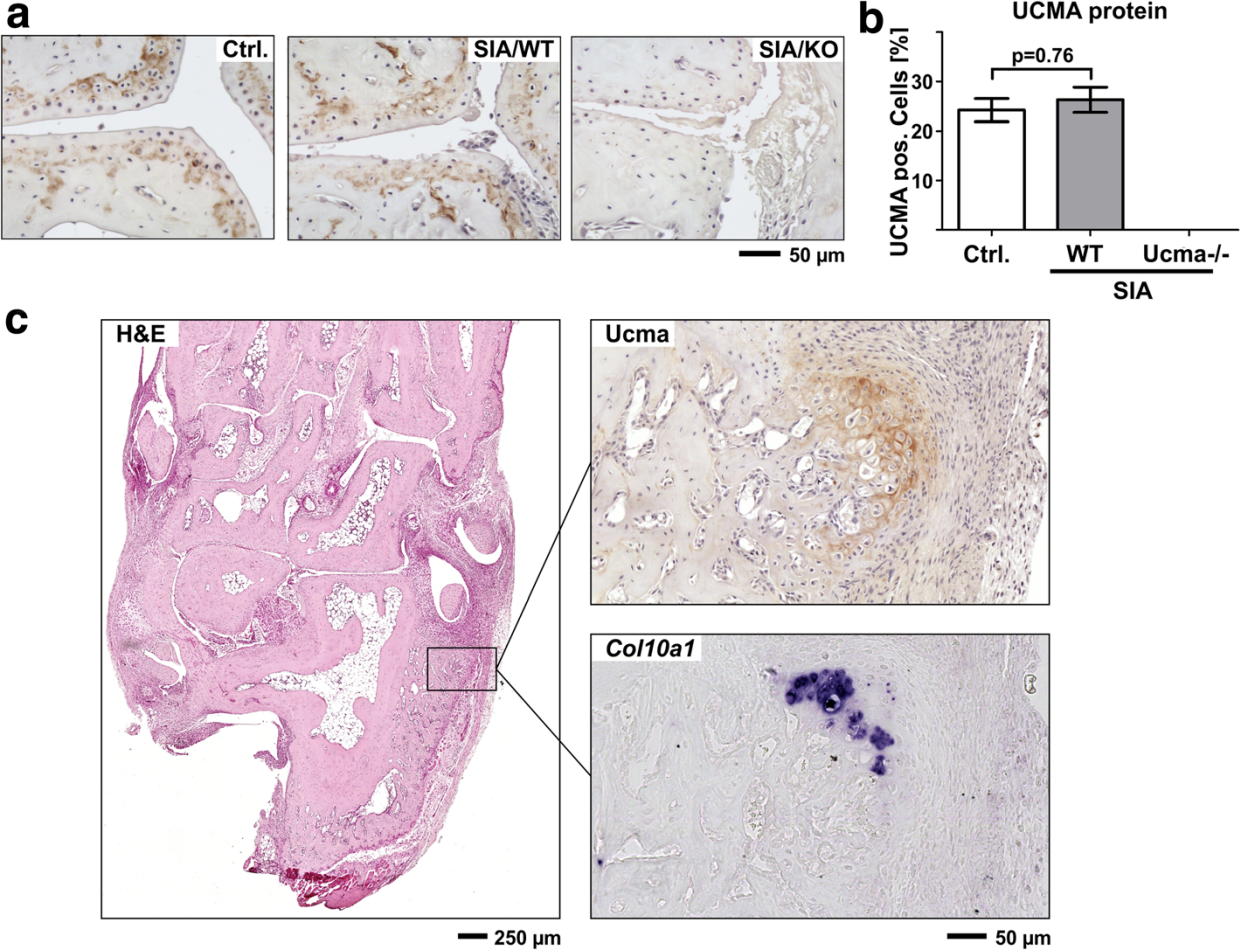
Ucma is expressed in the articular cartilage and in osteophytes during arthritis. **a** Representative immunohistochemical stainings of Ucma in articular cartilage of metatarsal joints from non-arthritic control mice (Ctrl.), and WT (SIA/WT) and Ucma-deficient (SIA/KO) mice with serum-induced arthritis (SIA). **b** Quantification of Ucma-positive chondrocytes (*n* = 5/group). Means ± SEM shown. **c** Representative immunohistochemical staining of Ucma on hind paw section from WT mouse with SIA shows Ucma protein in growing osteophyte at the calcaneus. H&E staining on a consecutive section shown for orientation. RNA in-situ hybridisation for *collagen10a1* (*Col10a1*) on a consecutive section indicates presence of hypertrophic chondrocytes adjacent to Ucma-positive chondrocytes at the calcaneus. **a, c** Composite images of 4–16 individual micrographs, respectively. H&E hematoxylin and eosin, KO knock-out, Ucma Upper zone of growth plate and cartilage matrix-associated protein, Ucma^−/−^ Ucma-deficient, WT wild type

This observation prompted us to investigate whether Ucma may not only play a role during degenerative joint diseases, such as OA, but also in inflammatory arthritis. Therefore, we compared Ucma protein expression in hind paws of WT mice with and without serum-induced arthritis (SIA). We observed significant Ucma protein expression in articular cartilage, which, however, was not altered in mice with experimental arthritis (Fig. 2a, b). In accordance, Ucma mRNA levels were not altered during SIA (Additional file 3). Yet, in contrast to healthy controls, mice with SIA exhibited substantial Ucma protein expression at sites of forming osteophytes (Fig. 2c). The main region of Ucma expression during osteophyte formation corresponded to chondrocytes adjacent to *Col10a1*-expressing hypertrophic chondrocytes (Fig. 2c).

### Ucma does not affect joint inflammation in experimental arthritis

**Fig. 3:**
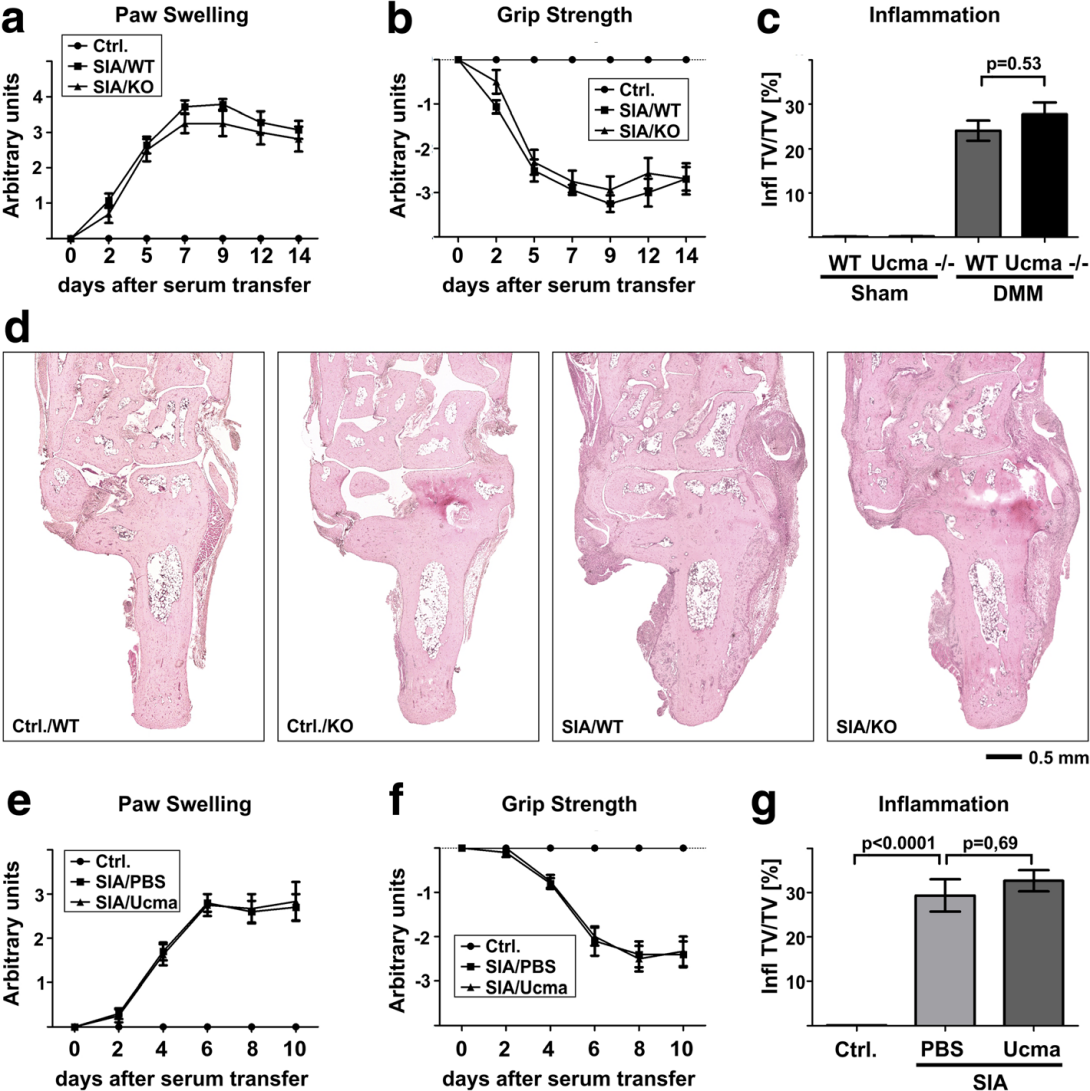
Ucma does not affect joint inflammation in experimental arthritis. **a–d** Wild-type (WT) and Ucma-deficient (KO; Ucma^−/−^) mice injected with K/BxN serum to induce arthritis (SIA) (day 0). Clinical signs of arthritis including paw swelling (**a**) and loss of grip strength (**b**) monitored for 14 days. **c** At day 14 mice were sacrificed and inflammation in the hind paw quantified by histomorphometry**. d** H&E stained histological sections: WT (Ctrl./WT) and Ucma-deficient (Ctrl./KO) non-arthritic controls; WT (SIA/WT) and Ucma-deficient (SIA/KO) mice with SIA. Means ± SEM shown; *n* = 7 or 8 per group. Composite images of 16–30 individual micrographs, respectively. **e–g** Ucma or vehicle (PBS) treatment in mice with serum-induced arthritis (SIA): (**e**) paw swelling, (**f**) grip strength and (**g**) inflammation 10 days after serum transfer. Means ±SEM shown; *n* = 3 or 5. Ctrl. control, Infl. TV/TV inflamed tissue volume per total tissue volume, KO knock-out, PBS phosphate-buffered saline, Ucma Upper zone of growth plate and cartilage matrix-associated protein

We next investigated whether Ucma may affect the severity of arthritis. Therefore, we studied the progression of SIA in WT and Ucma-deficient mice. To clinically assess the progression of arthritis, we monitored paw swelling and loss of grip strength in a semi-quantitative manner, in controls without induction of arthritis and in WT and Ucma-deficient mice induced for arthritis over 14 days. In contrast to controls, wild-type mice with SIA exhibited joint swelling (Fig. 3a) and loss of grip strength (Fig. 3b). Significant differences in these parameters between WT mice and their Ucma-deficient littermates, however, were not observed. Fourteen days after induction of arthritis, mice were sacrificed and inflamed paw tissue was quantified by histomorphometry. Synovitis was only detected in mice with SIA and its amount did not differ between WT and Ucma-deficient mice (Fig. 3c, d). To study the effect of excess Ucma on experimental arthritis, we systemically administered recombinant Ucma to C57/Bl6 mice with SIA. Systemic administration of Ucma was performed by daily intraperitoneal injections starting 1 day after serum transfer. Clinical signs of arthritis (i.e. paw swelling and loss of grip strength) were determined every other day. Ucma treatment did not affect these disease parameters (Fig. 3e, f). Likewise, histomorphometric quantification of inflammation on paw sections at 10 days after serum transfer did not reveal any effect of Ucma treatment on inflammation (Fig. 3g). Together, these observations indicate that Ucma does not affect joint inflammation.

### Ucma protects articular cartilage from arthritis-induced degradation

**Fig. 4:**
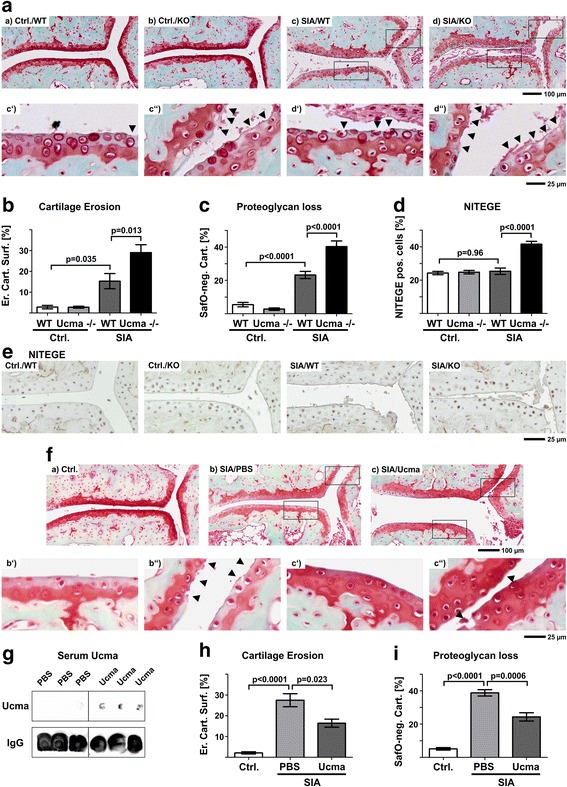
Arthritis-triggered cartilage is aggravated in Ucma-deficient mice but ameliorated after systemic treatment with recombinant Ucma. **A–E** Arthritis-triggered cartilage damage in WT and Ucma-deficient mice. Representative images of metatarsal joints on safranin-O stained hind paw sections. (c′, c″, d′, d″) Higher magnification inserts of (c) and (d), respectively. Arrowheads indicate eroded cartilage surface. **B** Quantification of eroded cartilage surface. **C** Proteoglycan loss in articular cartilage determined by quantification of safranin-O staining-negative articular cartilage (SafO-neg. Cart.). **D** Chondrocytes and their pericellular matrix stained positive for NITEGE neoepitope (NITEGE pos. cells). **E** Immunohistochemistry for ADAMTS-specific aggrecan cleavage products (NITEGE neoepitope). Means ± SEM shown; *n* = 7 or 8 per group. **F–I** Cartilage degeneration in mice with SIA treated with recombinant Ucma. **F** Representative images of safranin-O stained hind paw sections. (b′, b″, c′, c″) Higher magnification inserts of (b) and (c), respectively. Arrowheads indicate eroded cartilage surface. **G** Immunodetection of Ucma protein and immunoglobulin G (IgG, loading control) in serum by dot blot. **H** Cartilage erosion in metatarsal joints quantified on safranin-O stained sections of the hind paws. **I** proteoglycan loss by assessment of percentage of SafO-neg. Cart. Means ± SEM shown; *n* = 3 or 5 per group. **A**, **E**, **F** Composite images of 4–6 individual micrographs, respectively. ErCtrl./KO healthy control Ucma-deficient, Ctrl./WT healthy control WT, Cart. Surf. eroded cartilage surface, IgG immunoglobulin G, KO knock-out, NITEGE ADAMTS-specific aggrecan neoepitope, PBS phosphate-buffered saline, SIA serum-induced arthritis, SIA/KO Ucma-deficient with SIA, SIA/WT WT with SIA, Ucma Upper zone of growth plate and cartilage matrix-associated protein, Ucma^−/−^ Ucma-deficient, WT wild type

In order to investigate the impact of Ucma on arthritis-triggered cartilage damage, hind paw sections stained with safranin O were analysed for eroded cartilage surface and proteoglycan loss. As expected, healthy WT or mutant controls did not exhibit any overt cartilage damage in the metatarsal joints (Fig. 4A–C). During SIA, however, Ucma-deficient mice exhibited significantly increased levels of cartilage erosion (Fig. 4A, B) and proteoglycan loss (Fig. 4A, C) compared to WT littermates. This was not associated with significant Ucma-dependent differences in gene expression of *ADAMTS4* and *ADAMTS5* or *MMP3*, *MMP9* and *MMP13* (Additional file 3B–F). However, we observed significantly increased generation of ADAMTS-specific aggrecan degradation products with NITEGE neoepitope in the articular cartilage in arthritic Ucma-deficient mice compared to WT littermates (Fig. 4D, E). This finding indicates an increase in ADAMTS activity in Ucma-deficient mice with SIA, which is in line with Ucma-dependent inhibition of inflammation-triggered ADAMTS aggrecanase activity (Fig. 1) and with the increased proteoglycan loss in Ucma-deficient mice observed by safranin O staining in Fig. 4A.

In order to test, whether Ucma may consequently be a candidate for the treatment of arthritis-triggered cartilage damage in vivo, we investigated SIA-triggered cartilage degradation in C57/Bl6 mice systemically treated with recombinant Ucma. Ten days after serum transfer, Ucma was readily detectable in the serum of treated mice (Fig. 4G). Consistent with our loss-of-function data in Fig. 4A–E, mice treated with recombinant Ucma developed alleviated arthritis-triggered cartilage degeneration during SIA, determined as cartilage erosion (Fig. 4F, H) and proteoglycan loss (Fig. 4F, I) on safranin-O stained paw sections.

Together these data support the notion that Ucma protects from arthritis-triggered ADAMTS-dependent aggrecanolysis and cartilage degradation in SIA and propose Ucma as a candidate for future therapeutic strategies aiming at cartilage health during arthritis.

### Arthritic osteophyte formation is reduced in Ucma-deficient mice

**Fig. 5:**
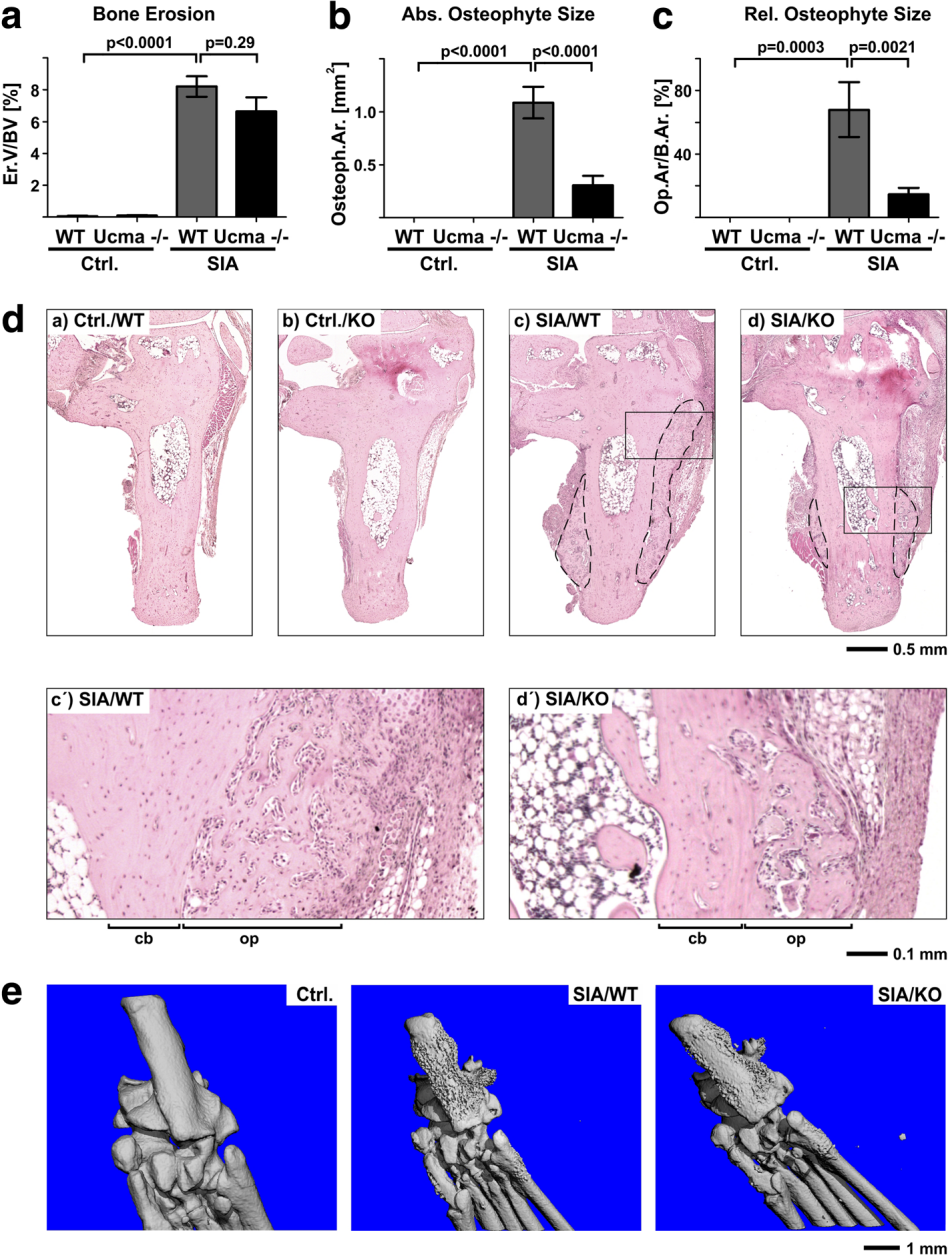
Reduced arthritis-triggered osteophyte formation in Ucma-deficient mice. Histomorphometry at 14 days after serum transfer showing (**A**) bone erosion (erosion volume per bone volume (Er.V/BV)), (**B**) maximal absolute osteophyte area (Osteoph.Ar.) and (**C**) relative osteophyte area expressed as osteophyte area per bone area (Op.Ar/B.Ar) in Ucma-deficient (Ucma^−/−^) and WT mice with serum-induced arthritis (SIA). **D** Representative micrographs of the calcaneus (H&E staining) in WT (Ctrl./WT) and Ucma-deficient (Ctrl./KO) non-arthritic controls and in WT (SIA/WT) and Ucma-deficient (SIA/KO) mice with SIA. Dashed line shows osteophytes. (b′, b″, c′, c″) Higher magnification inserts of (b) and (c), respectively. **E** Representative micro-CT scanning images (plantar view) of hind paws of WT control (Ctrl.), and WT (SIA/WT) and Ucma-deficient (SIA/KO) mice with SIA. Means ± SEM shown; *n* = 7 or 8 per group. **D** Composite images of 12–20 individual micrographs, respectively. cb normal cortical bone, KO knock-out, op osteophyte tissue, Ucma Upper zone of growth plate and cartilage matrix-associated protein, WT wild type

SIA is characterised by osteophyte formation and since Ucma was found to be expressed in chondrocytes within newly formed osteophytes we were interested in whether Ucma also influences osteophyte formation during arthritis. Therefore, the size of osteophytes at the calcaneus from WT and Ucma-deficient mice with SIA was measured histomorphometrically. Osteophytes in Ucma-deficient mice were substantially smaller than those in their WT littermates (Fig. 5B–D). The decrease in osteophyte size in Ucma-deficient arthritic mice was also detected by micro-CT scans (Fig. 5E). Although the treatment of WT mice with recombinant Ucma did not further increase the size of SIA-induced osteophytes 10 days after induction of arthritis (Additional file 4), these findings support the notion that Ucma promotes osteophyte formation. In contrast, arthritic bone erosion was not significantly affected by Ucma (Fig. 5A).

### Reduced osteoblast and osteoclast counts in hind paws of Ucma-deficient mice with SIA

**Fig. 6:**
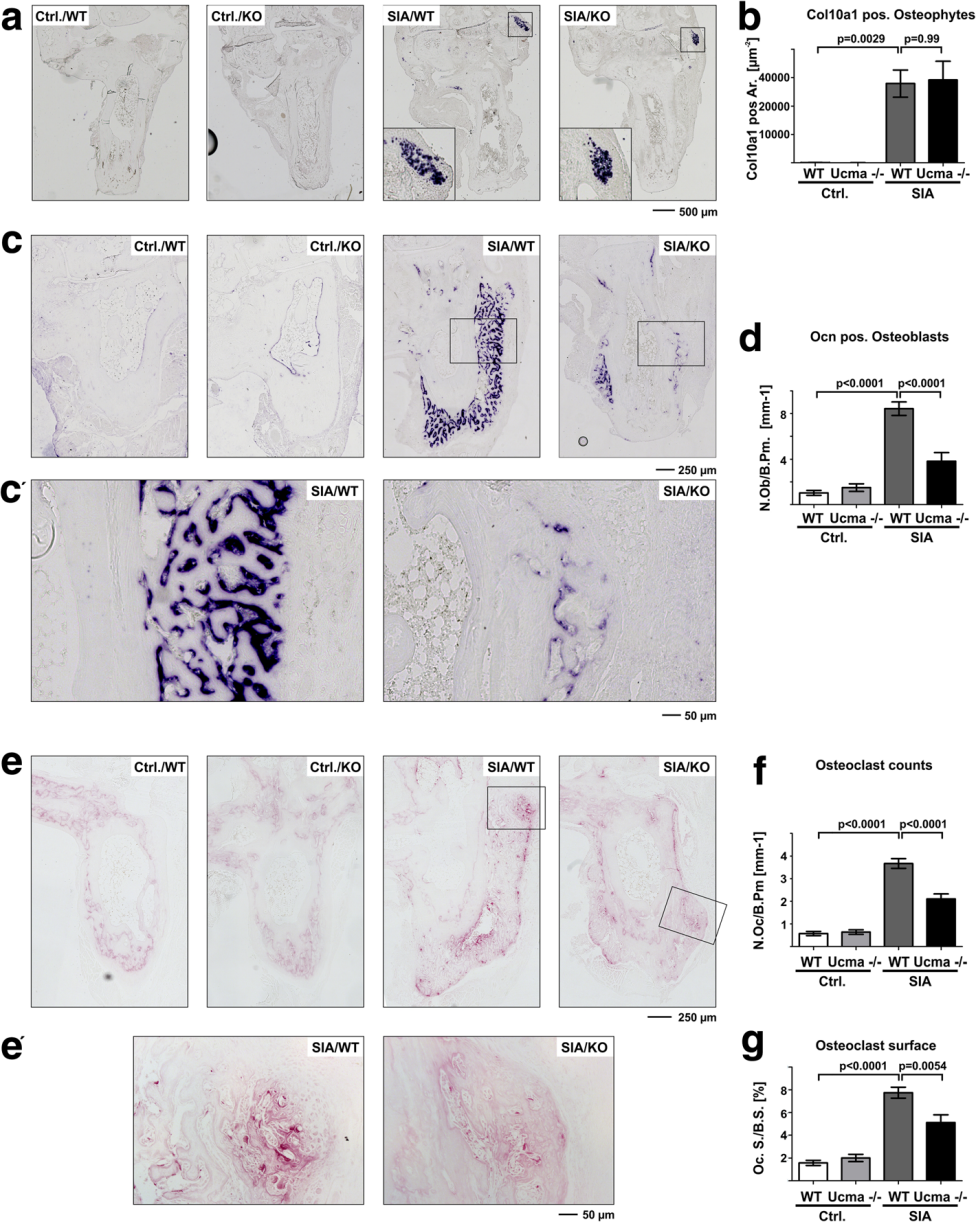
Reduced osteoblast and osteoclast numbers in arthritic Ucma-deficient mice. **a** RNA in-situ hybridisation for c*ollagen10a1* in hind paw sections of wild-type (WT) and Ucma-deficient (Ucma^−/−^) mice with serum-induced arthritis (SIA) Higher magnification inserts shown for mice with SIA. **b** Histomorphometric quantification of *collagen10a1* (*Col10a1*)-positive regions in osteophytes (maximal absolute c*ollagen10a1*-positive osteophyte area (Col10a1 pos. Ar.)). **c** RNA in-situ hybridisation for *osteocalcin* (*Ocn*). **c′** Inserts with higher magnification. **d** Histomorphometric quantification of Ocn-positive osteoblasts (osteoblast numbers per bone perimeter (N.Ob./B.Pm.)). **e** TRAP staining for osteoclasts. **e′** Inserts with higher magnification. **f** Histomorphometric quantification of TRAP-positive osteoclast numbers per bone perimeter in the hind paws (N.Oc./B.Pm). **g** Histomorphometric quantification of osteoclast surface/bone surface in the hind paws (Oc.S/B.S.). Means ± SEM shown; n = 7 or 8 per group. **a, c**, **e** Composite images of 12–20 individual micrographs, respectively. Ctrl./WT non-arthritic WT control, Ctrl./KO non-arthritic Ucma-deficient control, KO knock-out, SIA/WT WT with SIA, SIA/KO Ucma-deficient with SIA, Ucma Upper zone of growth plate and cartilage matrix-associated protein

To investigate the cell type mainly responsible for Ucma-dependent promotion of osteophyte formation, we investigated the cellular composition of hind paw bones from WT and Ucma-deficient mice with and without SIA. Since osteophytes form via a cartilage primordium, we first investigated the presence of hypertrophic chondrocytes by RNA in-situ hybridisation for *collagen10a1*. The *collagen10a1*-positive cells were detected at the margins of osteophytes at the calcaneus of mice with SIA. However, there was no apparent difference in hypertrophic chondrocytes between WT mice and their Ucma-deficient littermates (Fig. 6a, b).

Metabolically active osteoblasts were detected by RNA in-situ hybridisation for *osteocalcin*. As expected, the numbers of osteocalcin-positive osteoblasts considerably increased during SIA at the sites of osteophyte formation. In Ucma-deficient mice, however, substantially lower numbers of osteocalcin-positive osteoblasts were detected. Healthy WT and Ucma-deficient mice, however, did not differ in osteoblast numbers (Fig. 6c, d). In line with the reduction of metabolically active osteoblasts in osteophytes of Ucma-deficient mice, the number of metabolically active osteoclasts within the osteophytes was also significantly reduced (Fig. 6e–g). Hence, staining for TRAP activity showed lower osteoclast numbers in Ucma-deficient mice compared to WT littermates. *Receptor-activator of nuclear factor kappa B ligand* (*Rankl*; *tumour necrosis factor superfamily member 11* (*TNFSF11*)) or *osteoprotegerin* (*Opg*) mRNA expression, however, was not altered by Ucma (Additional file 3G,H). These data support our earlier findings that pathologic bone remodelling is reduced in Ucma-deficient mice [8].

## Discussion

This study identifies the cartilage-derived protein Ucma as a protective factor for cartilage loss in the context of inflammatory arthritis. Ucma physically binds to ADAMTS5 and inhibits its aggrecanase activity. Consequently, cartilage loss during arthritis is significantly more severe in Ucma-deficient mice than in WT mice but is alleviated when arthritic mice are treated with recombinant Ucma.

Aggrecanases of the ADAMTS family play a prominent role in cartilage destruction during pathological joint degeneration. They are considered to mediate one of the first steps in cartilage degradation by cleaving aggrecan within its interglobular domain, resulting in the release of the aggrecan chondroitin sulphate (CS)-rich region into the synovial fluid. This directly compromises joint function. Moreover, after the loss of the aggrecan CS-rich region, denuded collagen fibrils are more susceptible to collagenase cleavage and thereby ADAMTS aggrecanases contribute to further irreversible steps of cartilage degradation [3]. ADAMTS5 appears to be the primary aggrecanase, which is responsible for aggrecanolysis and cartilage degeneration in murine experimental osteoarthritis, while evidence rises for a contribution of both ADAMTS5 and ADAMTS4 in human cartilage degradation [2, 18–20].

Due to the pivotal role of these aggrecanases in cartilage degradation, their inhibition during joint pathologies is considered to be a primary aim in the treatment of cartilage degeneration. At least in murine models of arthritis, anti-aggrecanase regimens have provided promising results. Thus, intra-articular administration of monoclonal antibodies against ADAMTS5 has been shown to effectively ameliorate disease progression in a spontaneous mouse model of OA [21]. Other effective aggrecanase inhibitors have been shown to exert their inhibitory potential by physically biding to the thrombospondin type-1 repeats of ADAMTS4 and ADAMTS5. Thus, a truncated form of tissue inhibitor of metalloproteinases 3 (N-TIMP3) interacts with ADAMTS4 and ADAMTS5, thereby inhibiting their aggrecanase activity [22, 23]. Synthetic inhibitors of aggrecanases have also been designed to bind to ADAMTS4 and/or ADAMTS5 to inhibit their aggrecanase activity [3].

We have recently demonstrated that Ucma inhibits ADAMTS4 and ADAMTS5 aggrecanase activity in vitro and that Ucma-deficient mice develop a more severe cartilage damage in experimental osteoarthritis [8]. Here, we demonstrate that Ucma physically interacts with ADAMTS5. Moreover, recombinant Ucma inhibited ADAMTS5-triggered NITEGE formation in cultured 4C6 chondrocytes. These findings indicate that Ucma-dependent aggrecanase inhibition is directly mediated by physical interaction of Ucma with ADAMTS aggrecanases.

The pivotal role of ADAMTS aggrecanases in cartilage degradation is not confined to degenerative joint diseases such as osteoarthritis. ADAMTS aggrecanases are also key factors for cartilage degradation in inflammatory arthritis. Thus, Stanton et al. [2] have demonstrated that ADAMTS5-deficient mice are protected from cartilage degeneration during antigen-induced arthritis.

Consistently, our findings suggest that Ucma also protects from cartilage loss during inflammatory arthritis. The pro-inflammatory and arthritis-associated cytokine IL-1β is a potent inducer of ADAMTS activity in chondrocytes [24]. In line with this notion, IL-1β induced the formation of ADAMTS-specific aggrecan cleavage products (NITEGE neoepitope) in cultured 4C6 chondrocytes. Recombinant Ucma inhibited this IL-1β-triggered formation of NITEGE neoepitopes in 4C6 cultures, indicating that Ucma inhibits aggrecanase activity in cartilage under inflammatory conditions. Moreover, although *Adamts4* and *Adamts5* mRNA levels were not altered in Ucma-deficient mice, NITEGE neoepitope formation and cartilage damage were found to be significantly increased in articular cartilage of Ucma-deficient mice with SIA. Thus, these findings are consistent with the notion that Ucma is an inhibitor of ADAMTS-specific proteolytic cleavage of aggrecan also in inflammatory arthritis. Consequently, systemic administration of Ucma protected mice from cartilage degeneration during SIA.

These data are remarkable in the light that inflammation was no different between WT and Ucma-deficient mice or WT mice after Ucma treatment, which excludes indirect effects of Ucma on cartilage through potential down-regulation of inflammation. Synovial inflammation and clinical signs of arthritis were indistinguishable between WT and Ucma-deficient mice. Previous data by Cavaco et al. [25] indicated a possible anti-inflammatory potential of Ucma. Thus, recombinant Ucma reduced IL-1-induced gene expression of cyclooxygenase 2 (COX2) in human primary chondrocytes and synovial fibroblasts. Consequently, IL-1β-induced prostaglandin E2 (PGE2) secretion was decreased upon Ucma treatment [25]. The same group also observed that recombinant Ucma reduced TNFα and PGE2 production of LPS-stimulated macrophage-like cells [26]. In vivo, however, these effects seem to be of minor importance as the activity of SIA, which depends on cytokines such as IL-1 and TNFα, was not altered in the absence of Ucma or after systemic administration of recombinant Ucma.

While Ucma expression is strictly confined to the articular cartilage in steady-state conditions, additional expression of Ucma was also found in growing osteophytes in SIA, implying a possible role of Ucma also in SIA-related osteophyte formation or growth. In fact, SIA-triggered osteophyte formation was reduced in Ucma-deficient mice. Osteophyte formation usually resembles the process of endochondral ossification, where a cartilage primordium gets replaced by bone via an intermediate stage of calcified cartilage tissue rich in collagen type X [5]. Osteophytes in SIA are also formed via a cartilage primordium, which is reflected by the presence of *collagen10a1* expressing hypertrophic chondrocytes in developing osteophytes. Interestingly, however, we did not observe any Ucma-dependent difference in *collagen10a1*-positive hypertrophic chondrocytes at sites of osteophyte formation.

his finding may indicate that the formation and maturation of the cartilaginous osteophyte primordium is not affected by Ucma. The size of osteophytes, however, is not only determined by the modelling of the cartilage primordiae but also by its remodelling by osteoblasts and osteoclasts [5]. Intriguingly, reduced osteophyte formation in Ucma-deficient mice was associated with a decrease in osteoclasts and osteoblasts. Like in the case of experimental osteoarthritis [8], Ucma-dependent differences in osteoclast numbers were not associated with changes in *Rankl/Opg* mRNA ratios. This supports our earlier hypothesis that Ucma can stimulate osteoclast differentiation in a RANKL-independent manner. In fact, we demonstrated previously that Ucma can stimulate osteoclastogenesis in vitro and stimulation of pre-osteoclasts with Ucma rapidly induces the phosphorylation of MAP kinases, which may mediate such a RANKL-independent pro-osteoclastic effect [8, 27, 28]. Consistent with the observed decrease in osteoblast numbers in Ucma-deficient mice during SIA, Lee et al. [29] have previously reported that overexpression of Ucma in pre-osteoblasts promoted osteogenic differentiation in vitro. These data indicate that Ucma affects osteophyte formation on the level of bone remodelling rather than via regulating cartilage maturation.

While systemic administration of recombinant Ucma affected cartilage damage it did not elicit significant effects on osteophyte formation. This finding, however, may be explained by the earlier time point of histological analysis. Ucma-deficient mice and WT littermates have been histologically examined at 14 days after serum transfer, when osteophytes were fully developed. After Ucma administration the mice were analysed already 10 days after serum transfer, when osteophytes were still developing. This earlier time point was chosen for optimal detection of differences in cartilage degradation. However, as we have demonstrated, early stages of osteophyte development (formation of cartilage primordiae) do not appear to be affected by Ucma. Yet there may also be differences in the activities and local concentrations of endogenous and recombinant Ucma, which might also account for this, at first sight, unexpected result.

## Conclusions

Taken together, our results demonstrate a chondroprotective potential of Ucma during inflammatory arthritis. Ucma represents a cartilage-derived factor with regulatory functions on aggrecanases and the potential to limit cartilage degradation during inflammatory arthritis. In conjunction with anti-inflammatory treatment, Ucma treatment could therefore allow a better protection of the articular cartilage in arthritis.

## Additional files

Additional file 1: Primer sequences (DOCX 32 kb)

Additional file 2: Ucma physically interacts with ADAMTS5. Ucma–ADAMTS5 interactions investigated by slot blot binding assays: indicated amounts of recombinant ADAMTS5 blotted onto PVDF membrane incubated with recombinant FLAG-tagged Ucma (upper panel) or BSA (lower panel) and bound Ucma detected using rabbit anti-Ucma antibody (UCMA-1; 1:1000) and anti-rabbit IgG-HRP. Collagen II blotted as positive control. Representative data from two independent experiments (PDF 254 kb)

Additional file 3: Gene expression studies. **A–H** Gene expression in joint tissue from hind paws of non-arthritic controls (Ctrl.) and wild-type (WT) and Ucma-deficient (Ucma^−/−^) mice with serum-induced arthritis (SIA). Total RNA extracted from joint tissue from hind paws of respective mice. Gene expression analysed by real-time RT-PCR and normalised against cyclophilin A mRNA levels. Means ± SEM shown; *n* = 3 per group. ADAMTS A disintegrin-like and metalloproteinase with thrombospondin-1 motifs, MMP matrix metalloproteinase, Rankl receptor-activator of nuclear factor kappa B ligand, Opg osteoprotegerin (PDF 594 kb)

Additional file 4: Recombinant Ucma does not affect bone phenotype during SIA. SIA induced in WT C57/Bl6 mice by K/BxN serum transfer at day 0 and treated with daily i.p. injection of recombinant Ucma or carrier (PBS). Bone histomorphometry at 10 days after serum transfer revealed no effect of systemic Ucma administration on bone erosion (**A**), absolute and relative osteophyte size (**B, C**) or osteoclast numbers (**D**) and surface (**E**) in hind paws. Er.V/BV volume of bone erosion per total bone volume, Op.Ar. maximal absolute osteophyte area, Op.Ar/B.Ar. relative osteophyte area (osteophyte area per bone area), N.Oc./B.Pm osteoclast numbers per bone perimeter, Oc.S/B.S. osteoclast surface/bone surface. Means ± SEM shown; *n* = 3 or 5 per group (PDF 474 kb)

## Abbreviations

ADAMTS: A disintegrin-like and metalloproteinase domain with thrombospondin-1 repeats
ANOVA: Analysis of variance
BSA: Bovine serum albumin
COX: Cyclooxygenase
CS: Chondroitin sulphate
DMEM: Dulbecco’s modified Eagle medium
DMM: Destabilisation of the medial meniscus
H&E: Hematoxylin and eosin
HRP: Horseradish
i.p.: Intraperitoneal
IgG: Immunoglobulin G
IL-1: Interleukin-1
MAP: Mitogen-activated protein
micro-CT: Micro-computed tomography
MMP: Matrix metalloproteinase
OA: Osteoarthritis
OPG: Osteoprotegerin
PBS: Phosphate-buffered saline
PGE: Prostaglandin
PsA: Psoriasis arthritis
PVDF: Polyvinylidene difluoride
RA: Rheumatoid arthritis
RANKL: Receptor-activator of nuclear factor kappa B ligand
RT-PCR: Reverse transcriptase polymerase chain reaction
SEM: Standard error of the mean
SIA: Serum-induced arthritis
TIMP: Tissue inhibitor of metalloproteinases
TNF: Tumour necrosis factor
TRAP: Tartrate-resistant acid phosphatase
Ucma: Upper zone of growth plate and cartilage matrix-associated protein
WT: Wild type
