# Supplementary material for: Upper zone of growth plate and cartilage matrix associated protein protects cartilage during inflammatory arthritis
Source: Arthritis Res Ther. 2018 May 2;20:88. doi: 10.1186/s13075-018-1583-2 (PMC5932879; doi:10.1186/s13075-018-1583-2)
Supplement: Supplementary file 1 — Primer sequences (DOCX 32 kb) [file 13075_2018_1583_MOESM1_ESM.docx]

**Additional File 1: Primer sequences**

| **Gene** | **Primer** | **Sequence** | **Reference** |
| --- | --- | --- | --- |
| *Cyclophilin A* | forward | 5’-CCACCGTGTTCTTCGACAT-3’ | ([1](#_ENREF_1)) |
|  | reverse | 5’-CAGTGCTCAGAGCTCGAAAG-3’ |  |
| *Ucma* | forward | 5’-GTTTCTGGGGGTGCTCTGTA-3’ | ([1](#_ENREF_1)) |
|  | reverse | 5’- GAG GAA ATT GGA GGC ATC AG-3’ |  |
| *Rankl* | forward | 5’-AAACGCAGATTTGCAGGACT-3’ | ([2](#_ENREF_2)) |
|  | reverse | 5’-ACATCCAACCATGAGCCTTC-3’ |  |
| *Opg* | forward | 5’-AGCTGCTGAAGCTGTGGAA-3’ | ([3](#_ENREF_3)) |
|  | reverse | 5’-GGTTCGAGTGGCCGAGAT-3’ |  |
| *Adamts4* | forward | 5’- TGCAGGTAGAGGGTTTGACT-3’ | ([4](#_ENREF_4)) |
|  | reverse | 5‘- GACTCCGGATCTCCATTGAT-3‘ |  |
| *Adamts5* | forward | 5’- CCAGCAGTGCAACTTGACAT-3’ | ([4](#_ENREF_4)) |
|  | reverse | 5’- AGACACACCATTTGGCCTTG-3’ |  |
| *Mmp3* | forward | 5’- CGATGATGAACGATGGACAG-3’ | ([5](#_ENREF_5)) |
|  | reverse | 5‘- AGCCTTGGCTGAGTGGTAGA-3‘ |  |
| *Mmp9* | forward | 5’- CATTCGCGTGGATAAGGAGT-3’ | ([4](#_ENREF_4)) |
|  | reverse | 5’- TCACACGCCAGAAGAATTTG-3’ |  |
| *Mmp13* | forward | 5’-AAAGATTATCCCCGCCTCAT-3’ | ([6](#_ENREF_6)) |
|  | reverse | 5’-TGGGCCCATTGAAAAAGTAG-3’ |  |

**References**

1. Surmann-Schmitt C, Dietz U, Kireva T, Adam N, Park J, Tagariello A, et al. Ucma, a novel secreted cartilage-specific protein with implications in osteogenesis. J Biol Chem. 2008;283(11):7082-93.

2. Stock M, Menges S, Eitzinger N, Gesslein M, Botschner R, Wormser L, et al. A Dual Role of Upper Zone of Growth Plate and Cartilage Matrix-Associated Protein in Human and Mouse Osteoarthritic Cartilage: Inhibition of Aggrecanases and Promotion of Bone Turnover. Arthritis Rheumatol. 2017.

3. Palmqvist P, Lundberg P, Persson E, Johansson A, Lundgren I, Lie A, et al. Inhibition of hormone and cytokine-stimulated osteoclastogenesis and bone resorption by interleukin-4 and interleukin-13 is associated with increased osteoprotegerin and decreased RANKL and RANK in a STAT6-dependent pathway. J Biol Chem. 2006;281(5):2414-29.

4. Zwerina J, Redlich K, Polzer K, Joosten L, Kronke G, Distler J, et al. TNF-induced structural joint damage is mediated by IL-1. Proc Natl Acad Sci U S A. 2007;104(28):11742-7.

5. Barin JG, Baldeviano GC, Talor MV, Wu L, Ong S, Fairweather D, et al. Fatal eosinophilic myocarditis develops in the absence of IFN-gamma and IL-17A. J Immunol. 2013;191(8):4038-47.

6. Surmann-Schmitt C, Widmann N, Mallein-Gerin F, von der Mark K, Stock M. Stable subclones of the chondrogenic murine cell line MC615 mimic distinct stages of chondrocyte differentiation. J Cell Biochem. 2009;108(3):589-99.
