# Supplementary material for: Upper zone of growth plate and cartilage matrix associated protein protects cartilage during inflammatory arthritis
Source: Arthritis Res Ther. 2018 May 2;20:88. doi: 10.1186/s13075-018-1583-2 (PMC5932879; doi:10.1186/s13075-018-1583-2)
Supplement: Supplementary file 2 — Ucma physically interacts with ADAMTS5. Ucma–ADAMTS5 interactions investigated by slot blot binding assays: indicated amounts of recombinant ADAMTS5 blotted onto PVDF membrane incubated with recombinant FLAG-tagged Ucma (upper panel) or BSA (lower panel) and bound Ucma detected using rabbit anti-Ucma antibody (UCMA-1; 1:1000) and anti-rabbit IgG-HRP. Collagen II blotted as positive control. Representative data from two independent experiments (PDF 254 kb) [file 13075_2018_1583_MOESM2_ESM.pdf]

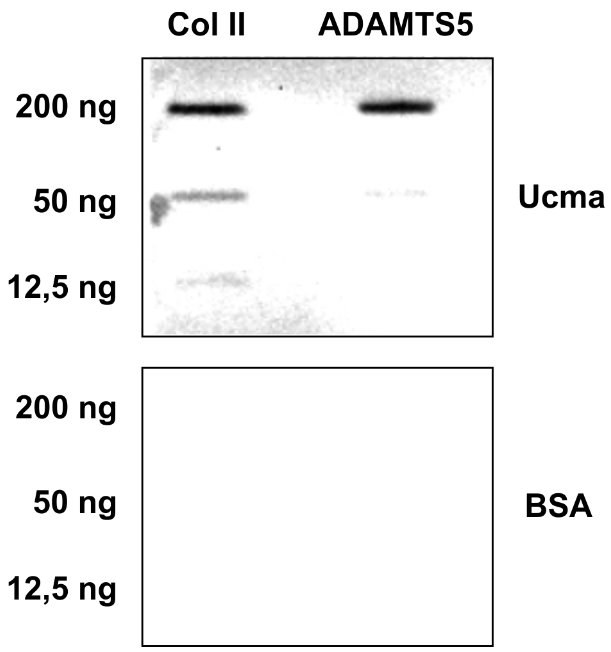

Additional File 2: Ucma physically interacts with ADAMTS5

Ucma-ADAMTS5 interactions were investigated by slot blot binding assays: indicated amounts of recombinant ADAMTS5 blotted onto a PVDF membrane were incubated with recombinant FLAG-tagged Ucma (upper panel) or BSA (lower panel) and bound Ucma was detected using an rabbit anti-Ucma antibody (UCMA-1; 1:1000) and anti-rabbit IgG-HRP. Collagen II was blotted as positive control. Representative data from 2 independent experiments are shown.
