# Supplementary material for: Upper zone of growth plate and cartilage matrix associated protein protects cartilage during inflammatory arthritis
Source: Arthritis Res Ther. 2018 May 2;20:88. doi: 10.1186/s13075-018-1583-2 (PMC5932879; doi:10.1186/s13075-018-1583-2)
Supplement: Supplementary file 3 — Gene expression studies. A–H Gene expression in joint tissue from hind paws of non-arthritic controls (Ctrl.) and wild-type (WT) and Ucma-deficient (Ucma−/−) mice with serum-induced arthritis (SIA). Total RNA extracted from joint tissue from hind paws of respective mice. Gene expression analysed by real-time RT-PCR and normalised against cyclophilin A mRNA levels. Means ± SEM shown; n = 3 per group. ADAMTS A disintegrin-like and metalloproteinase with thrombospondin-1 motifs, MMP matrix metalloproteinase, Rankl receptor-activator of nuclear factor kappa B ligand, Opg osteoprotegerin (PDF 594 kb) [file 13075_2018_1583_MOESM3_ESM.pdf]

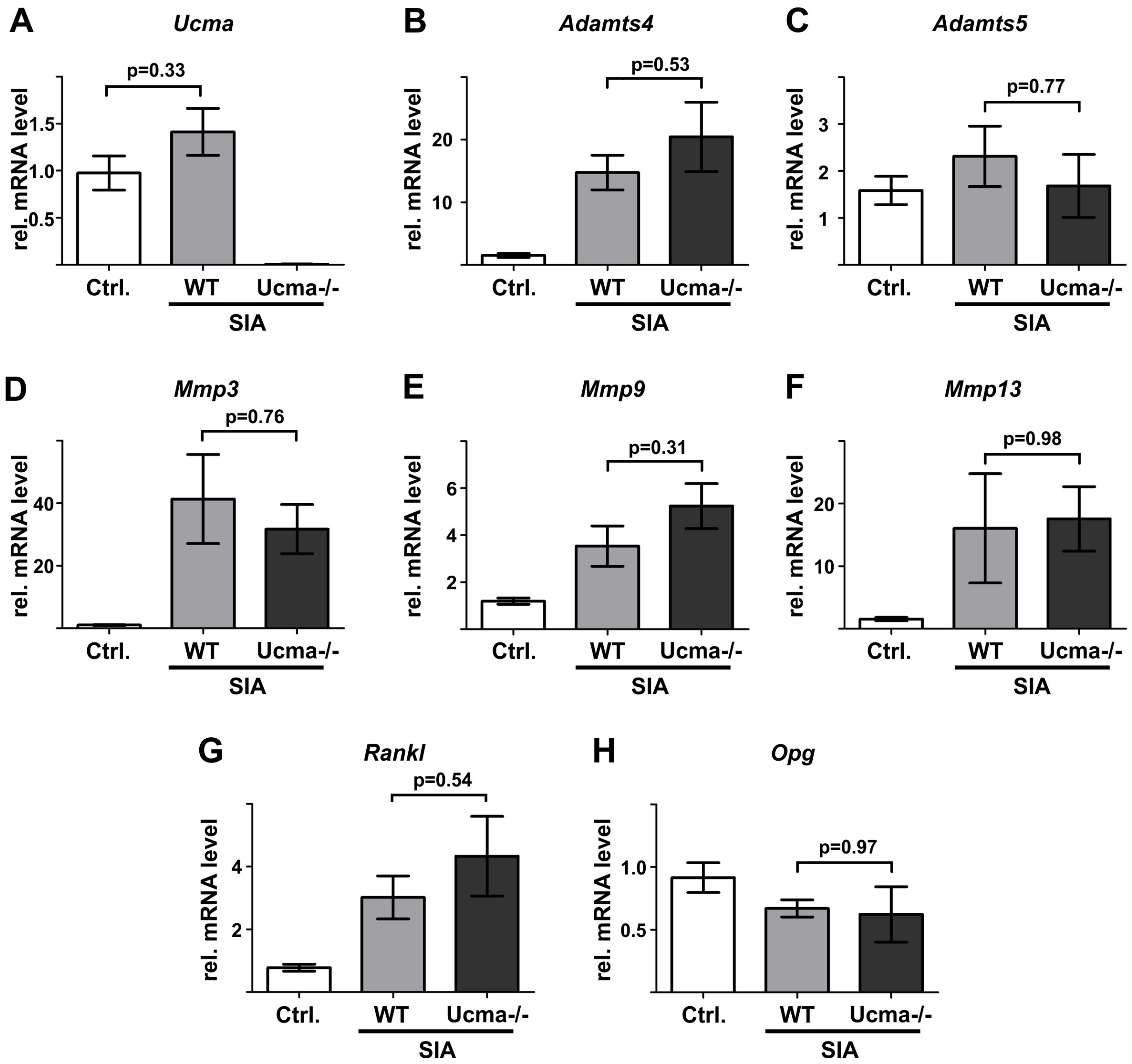

Additional File 3: Gene expression studies

A-H: Gene expression in joint tissue from the hind paws of non-arthritic controls (Ctrl.) and wild-type (WT) and Ucma-deficient mice (Ucma-/-) with serum-induced arthritis (SIA). Total RNA was extracted from joint tissue from the hind paws of respective mice. Gene expression was analysed by real-time RT-PCR and normalised against cyclophilin A mRNA levels. Means +/- SEM are shown; n=3 per group. Adamts, A Disintegrin-like and Metalloproteinase with Thrombospondin-1 motifs; Mmp, Matrix Metalloproteinase; Rankl, receptor-activator of nuclear factor kappa B ligand; Opg, osteoprotegerin.
