# Supplementary material for: Upper zone of growth plate and cartilage matrix associated protein protects cartilage during inflammatory arthritis
Source: Arthritis Res Ther. 2018 May 2;20:88. doi: 10.1186/s13075-018-1583-2 (PMC5932879; doi:10.1186/s13075-018-1583-2)
Supplement: Supplementary file 4 — Recombinant Ucma does not affect bone phenotype during SIA. SIA induced in WT C57/Bl6 mice by K/BxN serum transfer at day 0 and treated with daily i.p. injection of recombinant Ucma or carrier (PBS). Bone histomorphometry at 10 days after serum transfer revealed no effect of systemic Ucma administration on bone erosion (A), absolute and relative osteophyte size (B, C) or osteoclast numbers (D) and surface (E) in hind paws. Er.V/BV volume of bone erosion per total bone volume, Op.Ar. maximal absolute osteophyte area, Op.Ar/B.Ar. relative osteophyte area (osteophyte area per bone area), N.Oc./B.Pm osteoclast numbers per bone perimeter, Oc.S/B.S. osteoclast surface/bone surface. Means ± SEM shown; n = 3 or 5 per group (PDF 474 kb) [file 13075_2018_1583_MOESM4_ESM.pdf]

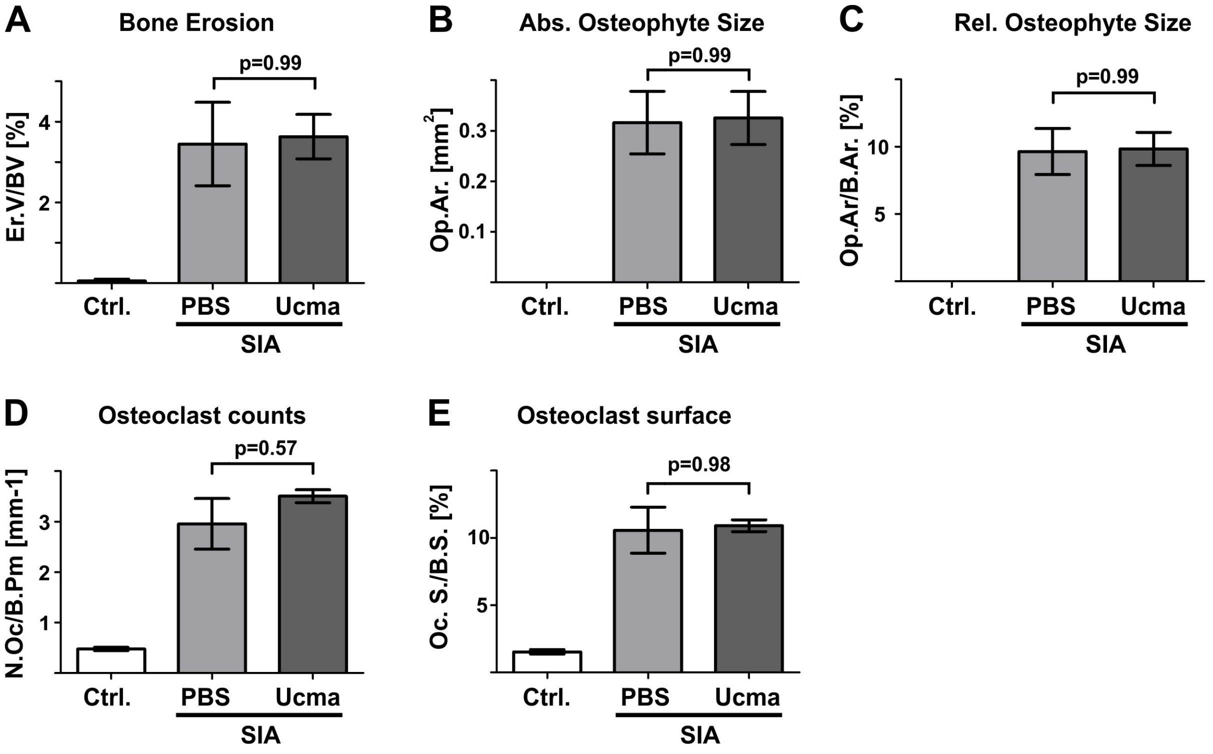

Additional File 4: Recombinant Ucma does not affect the bone phenotype during SIA

SIA was induced in WT C57/Bl6 mice by K/BxN serum transfer at day 0 and treated with a daily i.p. injection of recombinant Ucma or carrier (PBS). Bone histomorphometry at 10 days after serum transfer revealed no effect of systemic Ucma administration on bone erosion (A), absolute and relative osteophyte size (B, C) or osteoclast numbers (D) and surface (E) in the hind paws. Er.V/BV: Volume of bone erosion per total bone volume; Op.Ar.: maximal absolute osteophyte area; Op.Ar/B.Ar.: relative osteophyte area (osteophyte area per bone area). N.Oc./B.Pm: osteoclast numbers per bone perimeter; Oc.S/B.S.: osteoclast surface/bone surface. Means +/- SEM are shown; n=3 or 5 per group.
